# Mental disorders in a population sample with musculoskeletal disorders

**DOI:** 10.1186/1471-2474-7-37

**Published:** 2006-04-25

**Authors:** Scott B Patten, Jeanne VA Williams, JianLi Wang

**Affiliations:** 1 Department of Community Health Sciences, University of Calgary, 3330 Hospital Drive NW, Calgary, AB, T2N 4N1, Canada; 2 Department of Psychiatry, University of Calgary, 1403 – 29^th ^Street NW, Calgary, AB, T2N 2T9, Canada

## Abstract

**Background:**

Studies using clinical and volunteer samples have reported an elevated prevalence of mood disorders in association with rheumatoid arthritis and osteoarthritis. Clinical studies using anxiety rating scales have reported inconsistent results, but studies using diagnostic instruments have reported that anxiety disorders may be even more strongly associated with arthritis than is depression. One study reported an association between lifetime substance use disorders and arthritis.

**Methods:**

Data from iteration 1.2 of the Canadian Community Health Survey (CCHS) were used. This was a large-scale national Canadian health survey which administered the World Mental Health Composite International Diagnostic Interview to a sample of 36,984 subjects randomly selected from the national population. In the CCHS 1.2, subjects were asked whether they had been diagnosed by a health professional with arthritis or rheumatism.

**Results:**

Subjects reporting arthritis or rheumatism had an elevated prevalence of mood, anxiety and substance use disorders. The strength of association resembled that seen in an omnibus category reporting any chronic condition, but was weaker than that seen with back pain or fibromyalgia. The effect of arthritis or rheumatism interacted with age, such that the odds ratios became smaller with increasing age. Mood and anxiety disorders, along with arthritis or rheumatism made an independent contribution to disability.

**Conclusion:**

Arthritis is associated with psychiatric morbidity in the general population, and this morbidity is seen across a variety of mental disorders. The strength of association is consistent with that seen in persons with other self-reported medical conditions.

## Background

For people with rheumatoid arthritis and osteoarthritis, depression may be associated with pain sensitivity and with less effective coping with the illness. Zautra and Smith [1] used a measure of depressive symptom severity (the Mental Health Inventory) to assess the impact of depression in a volunteer sample with rheumatoid arthritis or osteoarthritis. Depressive symptoms in people with rheumatoid arthritis predicted elevated pain ratings, negative affect, negative life events, perceived stress and (decreased) positive event ratings. In osteoarthritis, depressive symptoms were found to predict higher levels of arthritis pain and negative affect. Anderson and others [2] used a depression adjective list to assess depressive symptom severity in a clinical sample with rheumatoid arthritis. They did not find that depression predicted pain severity, but found that it did predict observer-rated functional status.

Clinically diagnosed depressive disorders, as distinct from symptom ratings, and as defined by the American Psychiatric Association's Diagnostic and Statistical Manual of Mental Disorders (DSM-IV-TR) [3] are more directly related to clinical need than are symptom ratings. The first study to examine associations between arthritis and mental disorders in a community population was conducted by Wells and others [4,5] using data from one of five sites comprising the Epidemiologic Catchment Area studies, which were conducted in the United States in the 1980s. By epidemiological standards, the single site sample was not large, n = 3132 of whom 2554 were included in the analyses. Self-reported arthritis occurred with a frequency of18%, and a higher lifetime prevalence of affective, anxiety and substance use disorders were seen in this group than in subjects reporting no chronic conditions. Notably, this paper included a broad definition of substance use disorder – including both abuse and dependence on alcohol and other substances. The overall frequency of this category was 17.3% in subject without chronic conditions and 30.7% in subjects with arthritis [5]. An association was seen for lifetime, but not recent substance use disorders.

Baumeister & Härter conducted an investigation that integrated data from the German National Health Interview and Examination Survey, which used the Composite International Diagnostic Interview (CIDI), with data collected from inpatients with musculoskeletal conditions. They demonstrate a higher than expected prevalence of mood and anxiety disorders in the clinical subjects [6]. An elevated prevalence of substance use disorder was not found. McWilliams and others used data from another community study, the National Comorbidity Survey (NCS), to examine associations between "severe arthritis, rheumatism, or another bone or joint disease" and mood and anxiety disorders [7]. This study used the University of Michigan version of the CIDI to evaluate mental disorder prevalence. Both mood (major depression, dysthymia) and anxiety (generalized anxiety disorder, panic disorder, simple phobia, social phobia, agoraphobia and post-traumatic stress disorder) disorders had a higher prevalence in the arthritis, rheumatism or bone disease group than was seen in the general population. The strength of association was higher for anxiety than for mood disorders. This result was replicated subsequently using data from the Midlife Development in the United States Survey (MIDUS), which employed a brief predictive version of the CIDI and also included self-reported diagnoses of arthritis [8]. In the NCS analysis, a logistic regression model predicting disability was reported. Having arthritis, rheumatism or other bone or joint disease and having a psychiatric condition made an independent contribution to disability [7].

One record linkage study suggested that depressive episodes often precede completed suicides in people with rheumatoid arthritis and osteoarthritis, highlighting the potential clinical significance of depression in this population [9]. Another indication of clinical significance was reported by Löwe and others, who found that depression (in this study evaluated using the Patient Health Questionnaire [10]) contributed to disability in a way that was independent of severity of the rheumatologic disease [11]. This study was conducted in a clinical sample. In keeping with the idea that mental health is an important clinical issue for people with musculoskeletal conditions, Härter and others reported that rehabilitation patients with mental illness had diminished quality of life in several dimensions: general health, vitality, social and emotional role functioning and mental health [12].

One clinical study used DSM-IIIR criteria to diagnose major depression in a sample with rheumatoid and osteoarthritis who had screened high on a symptom rating scale [13], reporting a prevalence of 23% in rheumatoid arthritis and 10% in osteoarthritis. Finally, two clinical studies used structured diagnostic interviews to detect past episodes of major depression in clinical samples with rheumatoid arthritis. These studies reported lifetime prevalence estimates of 28.1% and 29.4% [14,15], approximately two to three times higher than reported general population lifetime prevalence. An extremely high prevalence of psychiatric caseness was reported in another clinical study of patients with rheumatoid arthritis, 39% [16]. However, because they used clinical samples, these studies may have overestimated prevalence by selecting particularly severely ill subjects.

Certain questions remain unanswered in the existing literature. First, although Wells and others [5] found an association between substance use disorders and arthritis, this finding has subsequently apparently not been replicated. Second, none of the existing studies have examined the possible association between musculoskeletal conditions and mania orbi polar disorder. Finally, the association between various conditions and disability should be confirmed in view of the result reported by McWilliams, that both anxiety and depressive disorders can make an independent contribution to disability in people with these disorders [7].

Some psychiatric epidemiological studies, including the Canadian Community Health Survey, Mental Health and Wellbeing (CCHS 1.2), have included high quality sampling and psychiatric diagnostic procedures. Unfortunately, suchstudies have typically incorporated limited information on conditions such as musculoskeletal disorders. For example, the CCHS 1.2 included only a single item asking each of its 36,984 respondents whether they had been diagnosed with "arthritis or rheumatism" by a health professional. Despite the lack of detail about rheumatologic status, such studies do provide an opportunity to describe the mental health status of people in the community who report having such disorders, and to answer questions that have not been addressed by the existing literature.

## Methods

The CCHS 1.2 was a nationally representative, community mental health survey conducted by Statistics Canada (Canada's national statistical agency) between May 2002 and December 2002. Detailed information about the methods employed in this study may be found in a paper by Gravel & Béland [17]. The target population included persons aged 15 years or over living in private occupied dwellings. Excluded were individuals living in health care institutions, on Indian Reserves, on government-owned land, in 1 of the 3 northern territories, or in remote regions. Full-time members of the armed forces were sampled separately, and are not included in the analyses reported here. One person aged 15 years or over was randomly selected from sampled households. A significant effort was made to interview respondents in person at their place of residence (86% of cases). Interviews were conducted in English, French, Chinese, or Punjabi (as required or requested by the interviewee).

From the initially selected 48,047 households, there was an 86.5% household-level response rate, and among responding household, there was an 89.0% person-level response rate. The overall response rate was thus 77.0%, resulting in a total sample size of 36,984 respondents.

The CCHS 1.2 interview was based on the WMH-CIDI [18], adapted for use in Canada. A copy of the Canadian adaptation is available on the Statistics Canada web page [19]. Trained lay interviewers using computer-assisted interviewing procedures administered the survey. Five disorders were evaluated: major depressive disorder, bipolar disorder, social phobia, agoraphobia, and panic disorder. Diagnostic algorithms followed DSM-IV criteria, with the exception of the duration requirement for a manic episode. The CCHS asked only whether manic symptoms had lasted "several days or longer" whereas a duration of 7 days is required by the DSM-IV when there has not been a need for hospitalization. In this analysis we differentiated between major depressive disorder and bipolar disorder by identifying subjects with one or more lifetime manic episodes according to the WMH-CIDI.

The Canadian adaptation of the WMH CIDI evaluated subjects for illicit drug dependence, and alcohol dependence was assessed in the survey using the CIDI Short Form [20]. In this analysis, substance dependence was defined as having either WMH-CIDI drug dependence, CIDI Short Form alcohol dependence, or both.

Subjects were also read a list of chronic medical conditions and asked whether they had been diagnosed with one of these conditions by a health professional. The exact wording of the item was: "Now I'd like to ask about certain chronic health conditions which you may have. We are interested in long-term conditions which have lasted or are expected to last 6 months or more and that have been diagnosed by a health professional." This was followed by: "Do you have arthritis or rheumatism, excluding fibromyalgia?" The accuracy of self-reported arthritis information has been shown to agree less closely with general practitioner reports than some other diagnoses. Kriegsman and others reported only a 72% concordance rate [21], but the questions used in the Kriedsman study did not require that the diagnosis be made by a health professional. A concordance rate of 86.9% for osteoarthritis and 96.1% for rheumatoid arthritis were reported by Barlow, Turner and Wright in a sample of rheumatology outpatients [22].

The CCHS 1.2 used a multistage, stratified cluster design to select eligible households. To correct the potential bias resulting from this complex survey design, Statistics Canada recommends a bootstrap procedure using a set of replicate weights that they supply. All results presented here were produced with this approach and are therefore representative of the targeted population. The standard error associated with specific estimates, p-values and confidence intervals (CIs), are also adjusted for survey design effects by the bootstrap weighting procedure. All analyses were conducted at the Prairie Regional Data Centre on the University of Calgary campus, using SAS software (8).

## Results

Of the 36,984 CCHS 1.2 participants, 8245 reported having arthritis or rheumatism (weighted prevalence 17.5%, 95% CI 17.0–18.0). This indicates that the vast majority of the sample did not have rheumatoid arthritis, which is generally thought to have a prevalence in the range of 0.5% to 1.0%. The estimate is very similar to that reported by Wells [5]. Among subjects reporting arthritis or rheumatism, the overall 12-month prevalence of major depressive disorder was 5.0% (95% CI 4.3 – 5.7). The 12-month prevalence of bipolar disorder was 1.2% (95% CI 0.9 – 1.5). We defined an aggregate category for panic disorder with or without agoraphobia, and which also included agoraphobia with panic-like symptoms, but not panic disorder. The 12-month prevalence of this category of anxiety disorders was 3.0% (95% CI 2.5 – 3.6). Diagnostic criteria for social phobia were met by 2.9% (95% CI 2.4 – 3.4). Substance dependence was present during the past year in 1.5% of subjects (95% CI 1.2 – 1.9).

These prevalence estimates are compared to those of subjects reporting back pain, and to subjects reporting fibromyalgia in Table 1. The table also includes a column for subjects reporting any one or more of the chronic conditions evaluated in the survey (other than osteoarthritis or rheumatism), and to subjects reporting no chronic conditions. For each psychiatric disorder, the prevalence was lower in subjects reporting arthritis or rheumatism than in those reporting back pain or fibromylagia. The prevalence figures closely resembled the aggregate category for subjects reporting one or more chronic conditions generally. The unadjusted prevalence of major depression, bipolar disorder and panic disorder was elevated in subjects reporting arthritis and rheumatism relative to this group. A logistic regression model was used to calculate unadjusted odd ratios by including only arthritis or rheumatism as a predictor variable. The unadjusted odds ratio for major depression was 1.4 (95% CI 1.1 – 1.6). For bipolar disorder the unadjusted odds ratio was 1.4 (1.0 – 1.9) and for panic disorder, 1.7 (95% CI 1.4 – 2.2). These results seem consistent with the pattern reported in the McWilliams paper, whereby panic disorder was among the conditions most strongly associated with severe arthritis, rheumatism or another bone or joint disease. [7]. However, social phobia was not significantly associated with arthritis or rheumatism in the unadjusted analysis (OR 1.0, 95% CI 0.8 – 1.2), nor was substance dependence more common in subjects reporting arthritis or rheumatism (OR 0.44, 95% CI 0.3 – 0.6). As these conditions are strongly associated with age and sex, however, adjusted estimates are more informative.

In a series of logistic regression models, the association between reported arthritis or rheumatism and each specific condition was explored. In each of these models, main effects for age and sex were significant, as expected. All of the psychiatric conditions tend to decline in prevalence with age in the general population. The mood and anxiety disorders were more common in women, whereas the opposite was true for substance use disorders. In each model, interaction terms were initially included, but were dropped if they were non-significant at the p = 0.05 level. The models pointed towards complex findings. Each model included at least some statistically significant interaction terms. Most importantly, age by reported arthritis or rheumatism interactions were observed in each model. This indicates that the strength of association varied with age. For major depression, the Wald statistic for the age by arthritis or rheumatism interaction was 7.41 (p = 0.006), for bipolar disorder it was 13.81 (p = 0.0002) and for substance dependence was 7.90 (p = 0.005). For social phobia, the model was further complicated by an age by sex by arthritis or rheumatism (3 way) interaction.

**Figure 1:**
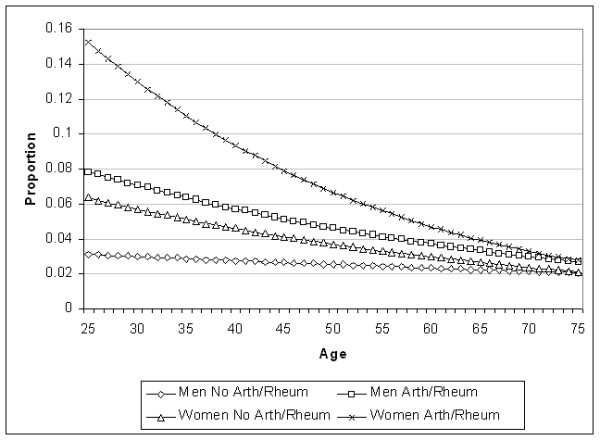
Fitted proportions: logistic regression model for major depression. The logistic equation is: ln(odds major depression = -3.22 + 1.33 (Arth/Rheum) - 0.0087 (Age) + 1.11 (female sex) - 0.014 (Arth/Rheum by Age) - 0.014 (Age by Sex). Fitted proportions: odds/1 + odds

**Figure 2:**
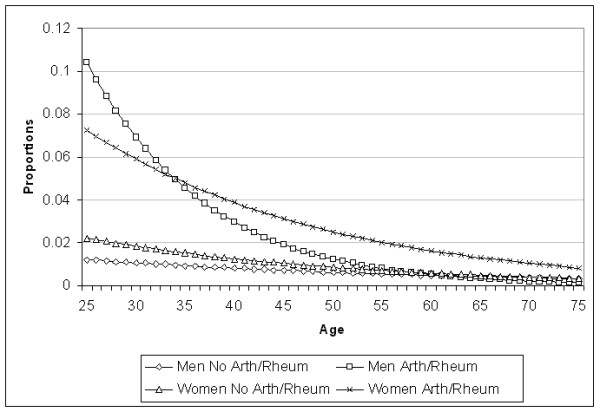
Fitted proportions: logistic regression model for bipolar disorder. The logistic equation is: -3.73 + 3.81 (Arth/Rheum) - 0.027 (Age) + 0.90 (Sex) - 0.063 (Arth/Rheum by Age) - 2.41 (Arth/Rheum by Sex) - 0.012 (Age by Sex) + 0.056 (Arth/Rheum by Age by Sex). Fitted proportions: odds/1 + odds

**Figure 3:**
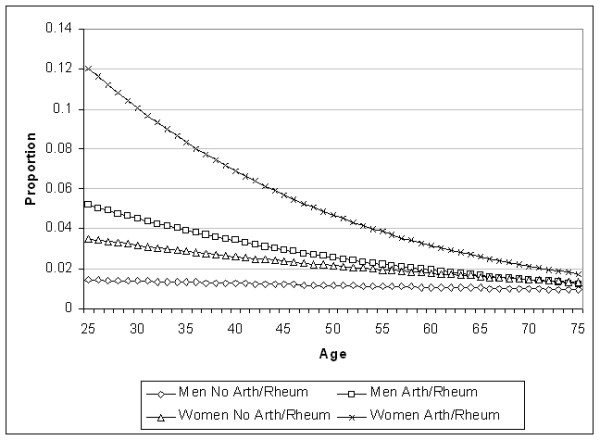
Fitted proportions: logistic regression model for panic disorder. The logistic equation is: -4.08 + 1.85 (Arth/Rheum) - 0.0084 (Age) + 1.21 (female sex) - 0.021 (Arth/Rheum by Age) -0.01164 (Age by Sex). Fitted proportions: odds/1 + odds

**Figure 4:**
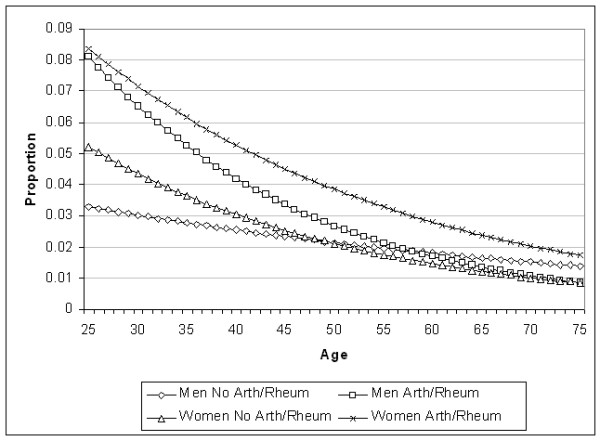
Fitted proportions: logistic regression model for social phobia. The logistic equation is: -2.93 + 1.67 (Arth/Rheum) - 0.018 (Age) + 0.97 (Sex) - 0.029 (Arth/Rheum by Age) - (Age by Sex) - 1.28 (Arth/Rheum by Sex) + 0.033 (Arth/Rheum by Age by Sex). Fitted proportions: odds/1 + odds

**Figure 5:**
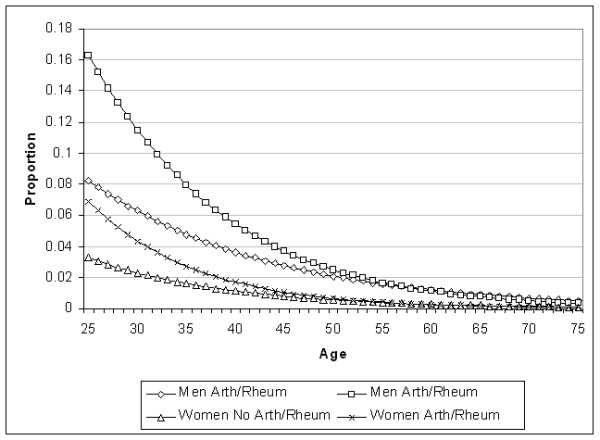
Fitted Proportions: Logistic Regression Model for Substance Dependence. The logistic equation is: -0.98 + 1.37842 (Arth/Rheum) - 0.057 (Age) - 0.56(Sex) - 0.024 (Arth/Rheum by Age) - 0.016 (Age by Sex). Fitted proportions: odds/1 + odds

The models all pointed towards strong effects in the young age groups. However, these cannot be interpreted as "main effects" for arthritis or rheumatism since interactions were present. In the presence of the interactions, the models indicate that there is no main effect, but rather that the association changes with advancing age. The logistic regression model for major depressive disorder is presented in Table 2. Fitted proportions for the major depression, bipolar disorder, panic disorder, social phobia and substance dependence models are presented in Figures 1, 2, 3, 4, 5.

Substance dependence was not independently associated with disability, but self-reported arthritis or rheumatism, mood disorders and anxiety disorders were. Logistic regression analyses found no interactions between these three sets of conditions, in other words, the effects were found to be multiplicative, confirming the result reported by McWilliams and others [7]. Arthritis or rheumatism, along with the mental disorders, made an independent contribution to disability. The logistic regression model is presented in Table 3.

## Discussion

One limitation of the study was the inability of the data collection interview (which consisted of a fully structured questionnaire administeredby trained lay interviewers) to distinguish specific rheumatologic diagnoses. While this is a limitation, its impact should not be overstated. Rheumatoid arthritis may not have a higher frequency of depressive disorders than osteoarthritis [13]. Pooling of homogeneous results from the literature using meta-analysis has been described as providing evidence of statistically significant differences (rheumatoid > osteoarthritis), but these were found to be small in magnitude [23].

A study by Hawley and Wolfe used the psychological scales in the Arthritis Impact Measurement Scale to assess anxiety and depression in subjects with a variety of rheumatologic conditions [24,25]. Although these authors used a symptom rating scale rather than a diagnostic measure, their findings were similar to those reported here. They reported that elevated levels of depression and anxiety were common, but that "fibrositis" and low back pain tended to have higher mean scores than rheumatoid and osteoarthritis. The results presented here also confirm the previous finding that psychiatric conditions and musculoskeletal disorders make an independent contribution to disability [7].

These results indicate that the mental health difficulties faced by people with rheumatologic diseases resemble those seen in other chronic conditions, at least in terms of their frequency. An association between bipolar disorder and arthritis or rheumatism has not previously been reported. This analysis did identify an association, but one of similar strength was seen in subjects reporting any chronic condition.

It appears that the nature of the association between mental disorder and arthritis or rheumatism is complex in the sense that age interactions appeared in logistic regression models. These indicated that the association may become weaker with advancing age. The general predictions of these models, however, were similar. Higher prevalences of the mental disorders were seen in younger age groups, and within age groups, higher prevalences were generally observed in those with arthritis or rheumatism. The diminishing odds ratios with increasing age may represent increasing resiliency, or may reflect clinical differences that exceeded the scope of this study to evaluate. For example, the prevalence of osteoarthritis increases progressively with age, such that the younger age groups may have a different mixture of rheumatoid and osteoarthritis, with a greater proportion having the latter. All previous community studies have had smaller sample sizes than this one. The interactions reported here may not have been identified in earlier studies because of lower statistical power.

The finding that substance use disorders are associated with arthritis, especially in younger age categories supports the Wells [5] report of an association. Interestingly, Wells found an association between arthritis and lifetime, but not recent, substance misuse. These authors speculated that the association between substance-use disorders and arthritis may manifest predominantly early in life. This idea seems consistent with the interaction between arthritis or rheumatism and age that was identified in this analysis.

McWilliams and others [7] reported a strong association between arthritis and post-traumatic stress disorder. This finding could not be replicated in the current study because this disorder was not assessed by the version of the CIDI employed in the CCHS 1.2. This is a limitation of the study, as is the lack of coverage of several other potentially relevant categories of disorder: personality disorders and somatoform disorders are probably the most important unmeasured categories. As noted above, self-reported diagnoses of musculoskeletal disorders may be inaccurate. Thedirection of bias that might have resulted from misclassification of arthritis status is difficult to anticipate. The most probable impact is non-differential misclassification bias, which is typically towards the null [26]. In support of the idea that misclassification is likely to be non-differential, Kriegsman and others [21] found that depression did not influence the extent of agreement between self-reported and physician diagnoses. The results presented here may therefore understate the strength of association.

As the data presented here were collected in a cross-sectional study, conclusions about causal effects cannot be directly supported. An elevated prevalence of mental disorders in people with musculoskeletal conditions could be due to an effect on prognosis as well as an effect on the risk of developing one of these disorders. However, these results have clinical implications for service delivery. Mental disorders are sufficiently frequent in this clinical group to suggest that case-finding measures, for example the routine administration of rating scales, may be useful strategies. This is particularly true in the younger age ranges, where high prevalences may translate into considerable predictive value from screening instruments having adequate sensitivity and specificity. Another implication is that such case-finding measures should not be restricted to depression, as a variety of mental disorders occur with an elevated frequency.

## Conclusion

The strength of association between a variety of mental disorders and self-reported arthritis or rheumatism resembles that of other chronic conditions in the general population. There is an elevated prevalence of mood, anxiety and substance use disorders. Both primary care and specialty care settings should have access to the resources necessary for managing these problems. The frequency of mental disorders and the strength of their association with arthritis and rheumatism was found to be highest in the youngest age categories, suggesting that formal case-finding or screening measures may be most fruitful in these age groups.

## List of Abbreviations

CCHS Canadian Community Health Survey Mental Health and Wellbeing

WMH CIDI World Mental Health Composite International Diagnostic Interview

CI Confidence Interval

## Competing interests

The author(s) have no competing interest to declare. The analyses reported here are based on data collected by Statistics Canada, the Canadian Government's statistical agency. The analysis itself does not reflect the opinions of Statistics Canada.

## Authors' contributions

Both Scott Patten and Jeanne Williams participated in conceptualization of the project, in preparation of the research proposal, in the analysis and preparation of the manuscript. Dr. Wang assisted with interpretation of the results and preparation of the manuscript.

## Pre-publication history

The pre-publication history for this paper can be accessed here:



## Figures and Tables

**Table 1:** Mental disorder prevalence, by reported musculoskeletal condition

	Arthritis or Rheumatism	Back Pain	Fibromyalgia	Any Chronic Condition***	No Chronic Condition
	% (95% CI)	% (95% CI)	% (95% CI)	% (95% CI)	% (95% CI)
Major	5.0	6.2	13.4	4.7	2.4
Depression	(4.3 – 5.7)	(5.4 – 6.9)	(8.9 – 17.9)	(4.3 – 5.1)	(2.1 – 2.8)

Bipolar	1.2	1.8	**	1.2	0.5
Disorder	(0.9 – 1.5)	(1.4 – 2.1)		(1.0 – 1.4)	(0.4 – 0.7)

Panic	3.0	3.6	5.2	2.6	1.0
Disorder*	(2.5 – 3.6)	(3.1 – 4.2)	(2.8 – 7.6)	(2.3 – 2.9)	(0.7 – 1.3)

Social	2.9	4.3	7.7	3.4	2.2
Phobia	(2.4 – 3.4)	(3.7 – 5.0)	(5.0 – 10.5)	(3.1 – 3.7)	(1.9 – 2.6

Substance	1.5	3.7	**	3.0	3.2
Dependence	(1.2 – 1.9)	(3.1–4.3)		(2.7 – 3.3)	(2.8 – 3.5)

* includes panic disorder with or without agoraphobia and agoraphobia without panic disorder

** estimates too imprecise to report, in keeping with Statistics Canada data release guidelines

*** any one or more of the following conditions: back pain, allergies to food and non-food, asthma, fibromyalgia, high blood pressure, migraine headaches, diabetes, heart disease, cancer, stomach/intestinal ulcers, effects of a stroke, bowel disorder/crohn's disease or colitis, cataracts, glaucoma, thyroid condition, chronic bronchitis, emphysema or COPD, epilepsy, multiple chemical sensitivies, chronic fatigue syndrome.

**Table 2:** Logistic regression model describing the association between major depressive disorder and arthritis/rheumatism, adjusting for demographic factors

	Coefficient	Odds Ratio	95% C.I.
Intercept	-3.225	--	--
Arthritis/Rheumatism	1.328	3.8	2.2 – 6.5
Age	-0.009	0.99	0.98 – 0.99
Female Sex	1.105	3.0	2.0 – 4.5
Arth/Rheum by Age Interaction	-0.014	0.99	0.98 – 0.99
Age by Sex Interaction	-0.014	0.99	0.98 – 0.99

**Table 3:** Logistic regression model predicting disability status* by disorder group and self-reported arthritis/rheumatism

	Coefficient	Odds Ratio	95% Confidence Interval
Intercept	-5.31	--	--
Arthritis/Rheumatism	1.17	3.2	2.6 – 4.0
Mood Disorder	1.44	4.2	3.3 – 5.4
Anxiety Disorder	0.77	2.2	1.6 – 2.9
Female Sex	0.21	1.2	1.0 – 1.5
Age 25–44	1.13	3.1	2.1 – 4.6
Age 45–64	1.71	5.6	3.6 – 8.4
Age 65 or Older	-0.52	0.6	0.3 – 1.1

* not working and reported reason for not working "disease or illness."
